# Dexamethasone reduces cell surface levels of CD11b on human eosinophils

**DOI:** 10.1080/09629359791505

**Published:** 1997-12

**Authors:** A. M. Das, L. H. K. Lim, R. J. Flower, M. Perretti

**Affiliations:** 1 Department of Biochemical Pharmacology The William Harvey Research Institute Charterhouse Square London EC1M 6BQ UK

## Abstract

Overnight incubation of human eosinophils (Eøs) with the glucocorticoid hormone dexamethasone (DEX; 0.1 μM) resulted in lower expression of the CD11b, but not CD49d, antigen on their plasma membrane, as assessed by flow cytometry. DEX produced a consistent inhibitory effect (ranging from 16% to 20%) when tested at a concentration of 0.1 μM. Eø stimulation with 100 ng/ml eotaxin produced an increase in CD11b (+26%), but not CD11c, levels and concomitantly a reduction (–25%) on CD62L expression. The inhibition exerted by DEX upon CD11b levels was also evident following eotaxin upregulation, with a degree of inhibition similar to that seen on basal levels. These data highlight a novel mechanism of action by which glucocorticoid hormones may be effective in reducing Eø accumulation during allergic inflammation in man.


Mediators of Inflammation, 6, 363–367 (1997)

### Dexamethasone reduces cell surface levels of CD11b on human eosinophils

A. M. Das[CA], L. H. K. Lim, R. J. Flower and M. Perretti

Department of Biochemical Pharmacology, The William Harvey Research Institute, Charterhouse Square, London EC1M 6BQ, UK

[CA]Corresponding Author
Tel: (+44) 171 982 6065
Fax: (+44) 171 982 6076
Email: a.m.das@mds.qmw.ac.uk

**Overnight incubation of human eosinophils (Eøs) with the glucocorticoid hormone dexamethasone (DEX; 0.1 μM) resulted in lower expression of the CD11b, but not CD49d, antigen on their plasma membrane, as assessed by flow cytometry. DEX produced a consistent inhibitory effect (ranging from 16% to 20%) when tested at a concentration of 0.1 μM. Eø stimulation with 100 ng/ml eotaxin produced an increase in CD11b (+26%), but not CD11c, levels and concomitantly a reduction (−25%) on CD62L expression. The inhibition exerted by DEX upon CD11b levels was also evident following eotaxin upregulation, with a degree of inhibition similar to that seen on basal levels. These data highlight a novel mechanism of action by which glucocorticoid hormones may be effective in reducing Eø accumulation during allergic inflammation in man.**

**Key words:** Integrins, Adhesion molecule, Eotaxin, Glucocorticoid hormones, CD18, CD62L, PAF, Mac-1, CD49d

### Introduction

Infiltration of eosinophils (Eøs) into sites of allergic inflammation is a characteristic feature of diseases such as asthma.[1] The products of activated Eøs have been shown to play a role in manifesting the symptoms of asthma.[2] Although many inflammatory mediators (including lipid mediators, cytokines and chemokines) are potent activators of Eøs, very few act specifically on this cell type. Eotaxin, which is a member of the CC family of chemokines, was isolated from the bronchoalveolar lavage after allergen challenge in sensitized guinea pigs.[3] It is a potent, and more importantly specific, activator of Eøs.

Activation of leukocytes by inflammatory mediators results in shedding of the selectin adhesion molecule CD62L from the cell surface and upregulation of the $\beta_2$ integrin CD11b/CD18.[4,5] This complex plays an important role in adhesive and activation processes of Eøs[6–8] and eotaxin increases levels of the CD11b subunit (Mac-1) on the cell surface of human Eøs.[9]

Glucocorticoid hormones are used routinely to treat allergic disorders, and indeed treatment with natural or synthetic glucocorticoids is effective in reducing the symptoms of these diseases, including asthma.[10] Although little is known about their mechanism of action, it appears that part of their anti-inflammatory actions may be mediated indirectly by reducing the number of circulating Eøs thereby attenuating Eø infiltration into allergic tissues.[11,12] More recently glucocorticoid hormones, in particular dexamathasone (DEX), have been shown to reduce adhesion molecule expression on selected target cells. For example, DEX was active on endothelial cells where levels of CD62E and CD54 were increased with cytokines or lipopolysaccharide.[13,14] Such an effect of DEX on the activated endothelium could contribute to the inhibition exerted by this drug on leukocyte accumulation during the host inflammatory response.[13] Much less is known about the effect of DEX upon adhesion molecules expressed by circulating leukocytes. A study with bovine neutrophils reported that administration of DEX *in vivo* reduced basal expression of CD62L and CD18 levels on this polymorphonuclear leukocyte.[15]

To date, no studies have investigated an effect of glucocorticoids on the adhesion molecules expressed on Eø plasma membrane. In the present study we have tested whether DEX affected either basal or eotaxin-upregulated CD11b levels on human Eøs.

### Material and Methods

### Isolation of human blood Eøs

Blood (80 ml) was obtained, out of the hay-fever season, from individuals who suffered

© 1997 Rapid Science Publishers from allergic rhinitis, and it was collected into sodium citrate. After dextran sedimentation (final concentration 1.2% dextran; Pharmacia, Uppsala, Sweden) the buffy coat was layered onto histopaque gradients (density 1.077; Sigma, Poole, UK). After centrifugation at $400 \times g$ for 25 min the leukocyte pellet was collected and all procedures were carried out at 4°C from this point. The remaining red blood cells in the granulocyte pellet were lysed hypotonically. Eøs were separated from neutrophils by incubation with anti-CD16 MACS microbeads (Miltenyi Biotec GmbH, Germany) at a concentration of 40 μl microbeads per $1 \times 10^8$ granulocytes followed by elution of the Eøs on a CS-type MACS column and magnetic separator (Milltenyi Biotec GmbH, Germany). The Eøs obtained were greater than 96% pure with contaminating cells constituting of mononuclear cells. Cells were either incubated overnight with DEX or stimulated with eotaxin immediately (see below).

### Overnight incubation of Eøs with DEX

Cells were resuspended at a concentration of $5 \times 10^6$ Eøs per ml in RPMI 1640 medium containing 10% heat-inactivated fetal bovine serum, 1% penicillin-streptomycin, 0.5% gentamycin and 2 mM L-glutamine (all obtained from Sigma, Poole, UK). One hundred μl of the cell suspension was added to flat-bottomed 96-well plates (in duplicate for each treatment). Either 100 μl buffer or different concentrations of DEX (1–0.001 μM final concentration; David Ball Laboratories, Warwick, UK) was added to the wells and the cells incubated at 37°C with 5% $CO_2$ in air for 18 h. After the incubation, the cells were washed and either (i) resuspended in phosphate buffered saline (PBS) containing 0.2% bovine serum albumin (BSA) for the measurement of cell surface adhesion molecules or, (ii) resuspended in RPMI 1640 containing 0.1% BSA for stimulation with mediators.

### Stimulation with mediators

Either fresh cells or cells which had been incubated overnight with DEX or buffer were incubated in a shaking water bath at 37°C for 30 min in the presence of buffer, human recombinant (hr) eotaxin (100 ng/ml final concentration; PeproTech EC Ltd, London, UK) or 0.1 μM final concentration platelet activating factor (PAF; C16 form, Sigma, Poole, UK). The reaction was stopped by placing the samples on ice for 5 min. The cells were then pelleted and resuspended in PBS containing 0.2% BSA for the measurement of cell surface adhesion molecules.

### Measurement of cell surface adhesion molecules

The presence of CD11b, CD11c, CD62L and CD49d ($\alpha_4$ subunit of the $\beta_1$ heterodimer CD49d/CD29) were measured on the cell surface using FACS analysis as previously described.[16] For this purpose, the following monoclonal antibodies (mAb) were used: mouse-anti human CD11b (clone 44; Serotec, Oxford, UK); mouse-anti human CD11c (clone 3.9; Serotec); mouse anti-human CD62L (clone FMC46; Serotec), and mouse anti-human CD49d (MAX68P; kindly gifted by Dr M. Robinson, Celltech Therapeutics, Slough, UK). MAX68P is a well characterized monoclonal antibody which has been shown to bind to Chinese hamster ovary cells transfected with CD49d (personal communication, Dr M. Robinson, Celltech Therapeutics). Briefly, cells were placed in flat-bottomed 96-well plates ($2.5 \times 10^5$ Eøs per well, 20 μl). Non-specific binding sites were blocked with 20 μl human IgG (15 mg/ml) before the addition of 20 μl of the appropriate mAb (giving a final concentration of 10–20 μg/ml). Control wells were incubated with similar amounts of mouse IgG (Sigma). Plates were incubated at 4°C for 1 h prior to washing and a further incubation with 40 μl of FITC-conjugated sheep anti-mouse IgG antibody (1:50 dilution of stock; Sigma, Poole, UK) for 30 min at 4°C. The plates were washed again and FACS analysis performed using a FACScan II analyser (Becton Dickinson, CA, USA) with air-cooled 100 mW argon ion laser tuned to 488 nm and Consort 32 computer running Lysis II software. The Eø population could be clearly identified because of the side and forward scatter characteristics (confirming in this way the high degree of purity) and the value of the mean fluorescence intensity (MFI) associated with it was measured at 548 nm with a band width of 10 nm (FL-1 channel). Results are reported as the MFI units for each sample and have been corrected for the control (mouse IgG) value.

### Statistics

Data are mean ± SEM of $n$ experiments performed in duplicate. Statistical analysis was performed on raw data using the Student's paired $t$-test.

### Results

### Effect of eotaxin on cell surface adhesion molecule levels

When human Eøs were incubated with eotaxin for 30 min, there was a significant increase (21%) in the levels of CD11b molecules on the cell surface with a concomitant 32% reduction in the CD62L levels (Fig. 1). In comparison, we found no effect on the levels of CD11c. We also examined the effect of another mediator, PAF, on the levels of CD11b and CD62L. From a typical experiment where the basal levels of CD11b was 220 MFI units, after eotaxin or PAF exposure this increased to 277 (26% increase) and 303 (38% increase), respectively. Similarly, basal levels of CD62L was 131 MFI, and this was reduced to 117 and 74 MFI units after eotaxin and PAF stimulation, respectively.

### Effect of DEX on basal and stimulated CD11b levels

Next, we investigated the effect of DEX on the levels of CD11b. For this series of experiments, Eøs were incubated for 18 h with or without the glucocorticoid hormone after which either (i) cell surface CD11b levels were measured or, (ii) a 30 min stimulation with eotaxin was performed prior to quantification of cell surface levels of CD11b. Incubation of Eøs overnight (in the absence of DEX) did not affect CD11b expression when compared to levels of the integrin on freshly isolated Eøs. Further, overnight incubation did not alter the extent of eotaxin-stimulated CD11b increase when compared to fresh Eøs (data not shown).

Following incubation with DEX there was a significant reduction on basal CD11b expression on Eø plasma membrane (Fig. 2). A similar reduction was also measured after Eø stimulation with eotaxin. The effect of DEX on basal CD11b levels was investigated further, finding a similar inhibition at all doses tested (a typical experiment is shown in the insert of Fig. 2). We finally investigated whether this effect of DEX was the result of a non-specific suppression of Eø genomic activity, or rather, was specific for CD11b. The data shown in Table 1 illustrates

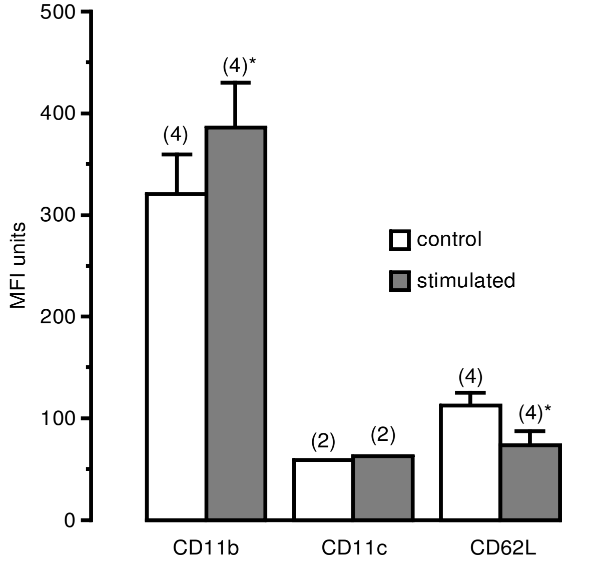

FIG. 1. The effect of eotaxin on Eø cell surface CD11b, CD11c and CD62L levels. Freshly isolated Eøs were incubated with either buffer (control) or 100 ng/ml hr eotaxin for 30 min at 37°C. Cell surface levels of the adhesion molecules were measured using FACS analysis. Data are mean ± SEM of (*n*) experiments performed in duplicate. *$P < 0.05$ when compared with appropriate control.

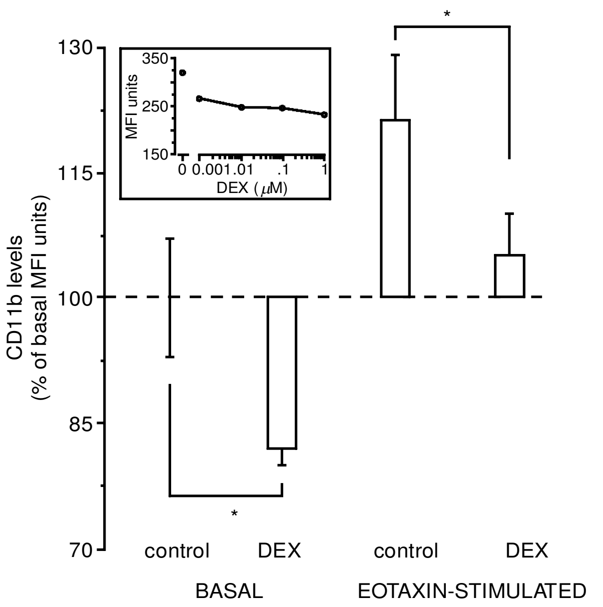

FIG. 2. The effect of DEX on basal or eotaxin-stimulated CD11b levels on Eøs. Eøs were incubated for 18 h with either buffer (control) or 0.1 μM DEX. Cell surface CD11b levels were measured either immediately (basal) or after the cells were incubated with either buffer or 100 ng/ml hr eotaxin for a further 30 min at 37°C. Basal value (100%) was 347 MFI units. Data are mean ± SEM from three to four experiments performed in duplicate. The insert shows a typical dose response to DEX on basal CD11b levels. *$P < 0.05$ when compared with control (calculated on original values).

**Table 1**. Effect of DEX on basal CD11b and CD49d levels on human Eøs

| Adhesion molecule | Control (MFI units) | DEX (MFI units) |
|---|---|---|
| CD11b ($n = 4$) | 347 ± 24 | 287 ± 25* |
| CD49d ($n = 5$) | 31 ± 6 | 36 ± 8 |

Eøs were incubated for 18 h with either buffer (control) or 0.1 μM DEX after which the cell surface levels of CD11b and CD49d were measured. Data are mean ± SEM of (*n*) experiments performed in duplicate. *$P < 0.05$ when compared with control values.

the lack of effect of DEX incubation on basal CD49d expression on the plasma membrane of Eøs.

### Discussion

In the present study we have investigated the effect of DEX on the expression of adhesion molecules on the surface of human Eøs. We examined the effect of DEX on basal, as well as eotaxin-stimulated, CD11b levels. We have shown, to our knowledge for the first time, that DEX is able to reduce the expression of CD11b on human Eøs.

We used the CC chemokine eotaxin to stimulate CD11b upregulation on Eøs. The concentration of eotaxin used was shown to be maximal for upregulating CD11b on Eøs in previous studies.[9,17] Different levels of CD11b upregulation has been achieved between studies. One reason for the difference could be the source of Eøs; a previous study used Eøs from non-atopic donors[9] whereas we have used Eøs from atopic subjects. Therefore, the Eøs in the present study might be desensitized to certain mediators through exposure *in vivo*. It also appears that the cells were more responsive to PAF than eotaxin which is in contrast to previous findings.[9] Again, this may be due to the desensitization status of the cells.

Together with cytokines and chemokines, adhesion molecules play a pivotal role in the process of leukocyte recruitment. Recently adhesion molecules have been shown to be a target (although not too sensitive) for glucocorticoid hormone action.[13] Whereas DEX effect on CD62E or CD54 upregulation on endothelial cells, fibroblasts and epithelial cells is well accepted, much less is known of the potential effect of this steroid on leukocyte adhesion molecules. Few studies have reported an ability of glucocorticoid hormones to reduce basal expression of selected adhesion molecules, though some species specificity has also been found. Prolonged (> 3 days) exposure to DEX reduced basal levels of CD62L and CD18 on bovine neutrophils;[15] CD62L and CD11a, but not CD11b, on rat neutrophils;[18] and CD54, but not CD11b, on rat monocytes.[16] In man, treatment with methyl prednisolone reduced upregulated, but not basal, CD11b expression on neutrophils after cardiopulmonary bypass, with no effect upon CD11a and CD11c.[19] No studies have addressed this question on Eøs.

In the present study DEX was found to reduce both basal CD11b expression on Eø cell surface, and this reduction explained the lower levels measured after cell activation with eotaxin. Therefore, the glucocorticoid affected the basal level of the antigen expressed on the Eø plasma membrane but not the cellular responsiveness to eotaxin. DEX effect was not due to an alteration of Eø viability. Further, it was not secondary to a non-specific suppression of Eø genomic activity as levels of a different integrin (CD49d) were not modified with Eø incubation with DEX. However, increased avidity, without alterations in total expression, of CD49d has been reported following exposure to chemokines.[20] It would therefore be of interest to investigate whether DEX exerted an effect on $\beta_1$ avidity states.

From these initial data we cannot identify the molecular mechanism by which DEX reduces CD11b expression on human Eøs. It could be the result of reduced gene transcription (which may be the case for CD54 as its promoter region contains glucocorticoid response elements (GRE)[21] or, an effect on mRNA or protein stability. The former hypothesis appears to be unlikely since GRE in the promoter region of the human CD11b gene has not been described.[22] However, an indirect mechanism of DEX through transcription factors or on CD11b vesicular turnover cannot be excluded.

Eø recruitment in experimental models of allergic inflammation relies on CD11b function as demonstrated *in vitro* on Eø transendothelial migration,[6] and *in vivo* on eotaxin-induced Eø accumulation.[23] Based on the data presented here, the possibility that DEX may affect Eø accumulation via, at least in part, an effect on CD11b should be taken into account, especially when long-lasting dose regimens are used. This can be particularly true for human diseases, where glucocorticoid hormones are given for prolonged periods for the treatment of asthmatic and allergic rhinitis.[10]

In conclusion, we believe the results from this study are important in understanding the anti-inflammatory mechanism(s) of actions exerted by glucocorticoid hormones in diseases of allergic inflammation.

### References

1. Seminario M-C, Gleich GJ. The role of eosinophils in the pathogenesis of asthma. *Curr Opinion Immunol* 1994; **6**: 860–864.
2. Frigas E, Motojima S, Gleich GJ. The eosinophils injury to the mucosa of the airways in the pathogenesis of bronchial asthma. *Eur Respir J* 1991; **4**: 123s–135s.
3. Jose PJ, Griffiths-Johnson DA, Collins PD, *et al.* Eotaxin: a potent eosinophil chemoattractant-cytokine detected in a guinea pig model of allergic airways inflammation. *J Exp Med* 1994; **179**: 881–887.
4. von Andrian UH, Chambers JD, McEvoy LM, Bargatze RF, Arfors K-E, Butcher EC. Two-step model of leukocyte-endothelial cell interaction in inflammation: distinct roles for LECAM-1 and the leukocyte β1 integrins in vivo. *Proc Natl Acad Sci USA* 1991; **88**: 7538–7542.
5. Crockett-Torabi E, Sulenbarger B, Smith CW, Fantone JC. Activation of human neutrophils through L-selectin and Mac-1 molecules. *J Immunol* 1995; **154**: 2291–2302.

6. Ebisawa M, Bochner BS, Georas SN, Schleimer RP. Eosinophil transendothelial migration induced by cytokines. I. Role of endothelial and eosinophil adhesion molecules in IL-1β-induced transendothelial migration. *J Immunol* 1992; **149**: 4021.
7. Hughes BJ, Hollers JC, Crockett-Torabi E, Smith CW. Recruitment of CD11b/CD18 to the neutrophil surface and adherence-dependent cell locomotion. *J Clin Invest* 1992; **90**: 1687–1696.
8. Horie S, Kita H. CD11b/CD18 (Mac-1) is required for degranulation of human eosinophils induced by human recombinant granulocyte-macrophage colony-stimulating factor and platelet-activating factor. *J Immunol* 1994; **152**: 5457–5467.
9. Tenscher K, Metzner B, Schopf E, Norgauer J, Czech W. Recombinant human eotaxin induces oxygen radical production, Ca2+-mobilization, actin reorganization, and CD11b upregulation in human eosinophils via a pertussis toxin-sensitive heterotrimeric guanine nucleotide-binding protein. *Blood* 1996; **88**: 3195–3199.
10. Pedersen S. Can steroids cause asthma to remit? *Am J Respir Crit Care Med* 1996; **153**: S31–S32.
11. Schleimer RP, Bochner BS. The effects of glucocorticoids on human eosinophils. *J Allergy Clin Immunol* 1994; **94**: 1202–1213.
12. Das AM, Flower RJ, Hellewell PG, Teixeira MM, Perretti M. A novel murine model of allergic inflammation to study the effect of dexamethasone on eosinophil recruitment. *Br J Pharmacol* 1997; **121**: 105–117.
13. Cronstein BN, Kimmel SC, Levin RI, Martiniuk F, Weissmann G. A mechanism for the antiinflammatory effects of corticosteroids: the glucocorticoid receptor regulates leukocyte adhesion to endothelial cells and expression of endothelial-leukocyte adhesion molecule 1 and intercellular adhesion molecule 1. *Proc Natl Acad Sci USA* 1992; **89**: 9991–9995.
14. Burke-Gaffney A, Hellewell PG. Regulation of ICAM-1 by dexamethasone in a human vascular endothelial cell line EA.hy926. *Am J Physiol* 1996; **270**: C552–C561.
15. Burton JL, Kehrli ME, Kapil S, Horst RL. Regulation of L-selectin and CD18 on bovine neutrophils by glucocorticoids: effects of cortisol and dexamethasone. *J Leukocyte Biol* 1995; **57**: 317–325.
16. Tailor A, Das AM, Getting SJ, Flower RJ, Perretti M. Subacute treatment of rats with dexamethasone reduces ICAM-1 levels on circulating monocytes. *Biochem Biophys Res Commun* 1997; **231**: 675–678.
17. Santamaria LF, Palacios JM, Beleta J. Inhibition of eotaxin-mediated human eosinophil activation and migration by the selective cyclic nucleotide phosphodiesterase type 4 inhibitor rolipram. *Br J Pharmacol* 1997; **121**: 1150–1154.
18. O'Leary EC, Marder P, Zuckerman SH. Glucocorticoid effects in an endotoxin-induced rat pulmonary inflammation model: differential effects on neutrophil influx, integrin expression, and inflammatory mediators. *Am J Respir Cell Mol Biol* 1996; **15**: 97–106.
19. Hill GE, Alonso A, Thiele GM, Robbins RA. Glucocorticoids blunt neutrophil CD11b surface glycoprotein upregulation during cardiopulmonary bypass in humans. *Anesth Analg* 1994; **79**: 23–27.
20. Weber C, Kitayama J, Springer TA. Differential regulation of β1 and β2 integrin avidity by chemoattractants in eosinophils. *Proc Natl Acad Sci USA* 1996; **93**: 10939–10944.
21. Caldenhoven E, Liden J, Wissink S, *et al.* Negative cross-talk between RelA and the glucocorticoid receptor: a possible mechanism for the antiinflammatory action of glucocorticoids. *Mol Endocrinol* 1995; **9**: 401–412.
22. Hickstein DD, Baker DM, Gollahoon KA, Back AL. Identification of the promoter of the myelomonocytic leukocyte integrin CD11b. *Proc Natl Acad Sci USA* 1992; **89**: 2105–2109.
23. Das AM, Flower RJ, Perretti M. Eotaxin-induced eosinophil migration in the peritoneal cavity of ovalbumin-sensitized mice. Mechanism of action. *J Immunol* 1997; **159**: 1466–1473.

ACKNOWLEDGEMENTS. This work was supported by the Wellcome Trust; R.J.F. is a principal fellow of the Wellcome Trust and M.P. is a postdoctoral fellow of the Arthritis and Rheumatism Council. The authors would like to thank Drs M. Robinson and A. Shock for helpful discussions.

**Received 4 August 1997;**
**accepted in revised form 29 August 1997**

## References

[PDF_00491] Burke-Gaffney A., Hellewell P. G. (1996). Regulation of ICAM-1 by dexamethasone in a human vascular endothelial cell line EAhy926.. Am J Physiol.

[PDF_00494] Burton J. L., Kehrli M. E., Kapil S., Horst R. L. (1995). Regulation of L-selectin and CD18 on bovine neutrophils by glucocorticoids: effects of cortisol and dexamethasone.. J Leukoc Biol.

[PDF_00456] Crockett-Torabi E., Sulenbarger B., Smith C. W., Fantone J. C. (1995). Activation of human neutrophils through L-selectin and Mac-1 molecules.. J Immunol.

[PDF_00485] Cronstein B. N., Kimmel S. C., Levin R. I., Martiniuk F., Weissmann G. (1992). A mechanism for the antiinflammatory effects of corticosteroids: the glucocorticoid receptor regulates leukocyte adhesion to endothelial cells and expression of endothelial-leukocyte adhesion molecule 1 and intercellular adhesion molecule 1.. Proc Natl Acad Sci U S A.

[PDF_00521] Das A. M., Flower R. J., Perretti M. (1997). Eotaxin-induced eosinophil migration in the peritoneal cavity of ovalbumin-sensitized mice: mechanism of action.. J Immunol.

[PDF_00461] Ebisawa M., Bochner B. S., Georas S. N., Schleimer R. P. (1992). Eosinophil transendothelial migration induced by cytokines. I. Role of endothelial and eosinophil adhesion molecules in IL-1 beta-induced transendothelial migration.. J Immunol.

[PDF_00518] Hickstein D. D., Baker D. M., Gollahon K. A., Back A. L. (1992). Identification of the promoter of the myelomonocytic leukocyte integrin CD11b.. Proc Natl Acad Sci U S A.

[PDF_00508] Hill G. E., Alonso A., Thiele G. M., Robbins R. A. (1994). Glucocorticoids blunt neutrophil CD11b surface glycoprotein upregulation during cardiopulmonary bypass in humans.. Anesth Analg.

[PDF_00468] Horie S., Kita H. (1994). CD11b/CD18 (Mac-1) is required for degranulation of human eosinophils induced by human recombinant granulocyte-macrophage colony-stimulating factor and platelet-activating factor.. J Immunol.

[PDF_00465] Hughes B. J., Hollers J. C., Crockett-Torabi E., Smith C. W. (1992). Recruitment of CD11b/CD18 to the neutrophil surface and adherence-dependent cell locomotion.. J Clin Invest.

[PDF_00449] Jose P. J., Griffiths-Johnson D. A., Collins P. D., Walsh D. T., Moqbel R., Totty N. F., Truong O., Hsuan J. J., Williams T. J. (1994). Eotaxin: a potent eosinophil chemoattractant cytokine detected in a guinea pig model of allergic airways inflammation.. J Exp Med.

[PDF_00504] O'Leary E. C., Marder P., Zuckerman S. H. (1996). Glucocorticoid effects in an endotoxin-induced rat pulmonary inflammation model: differential effects on neutrophil influx, integrin expression, and inflammatory mediators.. Am J Respir Cell Mol Biol.

[PDF_00477] Pedersen S. (1996). Can steroids cause asthma to remit?. Am J Respir Crit Care Med.

[PDF_00500] Santamaria L. F., Palacios J. M., Beleta J. (1997). Inhibition of eotaxin-mediated human eosinophil activation and migration by the selective cyclic nucleotide phosphodiesterase type 4 inhibitor rolipram.. Br J Pharmacol.

[PDF_00479] Schleimer R. P., Bochner B. S. (1994). The effects of glucocorticoids on human eosinophils.. J Allergy Clin Immunol.

[PDF_00444] Seminario M. C., Gleich G. J. (1994). The role of eosinophils in the pathogenesis of asthma.. Curr Opin Immunol.

[PDF_00497] Tailor A., Das A. M., Getting S. J., Flower R. J., Perretti M. (1997). Subacute treatment of rats with dexamethasone reduces ICAM-1 levels on circulating monocytes.. Biochem Biophys Res Commun.

[PDF_00472] Tenscher K., Metzner B., Schöpf E., Norgauer J., Czech W. (1996). Recombinant human eotaxin induces oxygen radical production, Ca(2+)-mobilization, actin reorganization, and CD11b upregulation in human eosinophils via a pertussis toxin-sensitive heterotrimeric guanine nucleotide-binding protein.. Blood.

[PDF_00511] Weber C., Kitayama J., Springer T. A. (1996). Differential regulation of beta 1 and beta 2 integrin avidity by chemoattractants in eosinophils.. Proc Natl Acad Sci U S A.

[PDF_00452] von Andrian U. H., Chambers J. D., McEvoy L. M., Bargatze R. F., Arfors K. E., Butcher E. C. (1991). Two-step model of leukocyte-endothelial cell interaction in inflammation: distinct roles for LECAM-1 and the leukocyte beta 2 integrins in vivo.. Proc Natl Acad Sci U S A.

